# Secondary sclerosing cholangitis in patients suffering cardiogenic shock

**DOI:** 10.1002/ehf2.15248

**Published:** 2025-02-26

**Authors:** Hugo Lanz, Clemens Scherer, Philipp Kasper, Christoph Adler, Leonhard Binzenhöfer, Sabine Hoffmann, Julia Höpler, Marie Kraft, Nils Gade, Raúl Nicolás Jamin, Ruben Evertz, Daniel Hoyer, Jörn Tongers, Christian Schulze, Christian Jung, Julia Claus, Janine Pöss, Lisa Crusius, Norman Mangner, Christian Hagl, Georg Nickenig, Sebastian Zimmer, Steffen Massberg, Holger Thiele, Franz Haertel, Enzo Lüsebrink

**Affiliations:** ^1^ Medizinische Klinik und Poliklinik I, Klinikum der Universität München, Munich, Germany and DZHK (German Center for Cardiovascular Research), partner site Munich Heart Alliance Munich Germany; ^2^ Klinik für Gastroenterologie und Hepatologie Universitätsklinikum Köln Köln Germany; ^3^ Klinik für Kardiologie, Angiologie, Pneumologie und internistische Intensivmedizin, Klinik III für Innere Medizin, Herzzentrum, Universität zu Köln Köln Germany; ^4^ Institute for Medical Information Processing, Biometry, and Epidemiology, Ludwig‐Maximilians Universität München Munich Germany; ^5^ Medizinische Klinik und Poliklinik II Universitätsklinikum Bonn Bonn Germany; ^6^ Department of Cardiology and Pneumology University of Göttingen Medical Center Göttingen Germany; ^7^ Universitätsklinik und Poliklinik für Innere Medizin III Kardiologie, Angiologie und Internistische Intensivmedizin, Universitätsklinikum Halle (Saale) Halle (Saale) Germany; ^8^ Klinik für Innere Medizin I Universitätsklinikum Jena Jena Germany; ^9^ Division of Cardiology, Pulmonology, and Vascular Medicine, Medical Faculty University Hospital Düsseldorf, Heinrich‐Heine‐University Düsseldorf Düsseldorf Germany; ^10^ Department of Internal Medicine/Cardiology, Leipzig Heart Institute Heart Center Leipzig at University of Leipzig Leipzig Germany; ^11^ Klinik für Innere Medizin und Kardiologie Herzzentrum‐Dresden an der Technischen Universität Dresden Dresden Germany; ^12^ Herzchirurgische Klinik und Poliklinik Klinikum der Universität München Munich Germany; ^13^ DZHK (German Center for Cardiovascular Research), partner site Munich Heart Alliance Munich Germany

**Keywords:** Cardiogenic shock, Secondary sclerosing cholangitis, Hepatic dysfunction

## Abstract

**Aims:**

Cardiogenic shock (CS) patients suffer from severe organ hypoperfusion, yet the incidence of secondary sclerosing cholangitis in critically ill patients (SSC‐CIP) in CS is poorly described. Given the limited evidence and severity of this syndrome, we aimed to further investigate SSC‐CIP in the context of CS.

**Methods and results:**

24 251 total CS patients admitted between 1 January 2010 and 31 December 2023 were retrospectively screened for the diagnosis of SSC‐CIP across nine German tertiary care centers. Following identification of confirmed SSC‐CIP diagnosis, baseline characteristics, laboratory values, SSC‐CIP‐specific imaging, diagnostics, and outcomes were obtained for analysis. 35 CS patients with a diagnosis of SSC‐CIP were identified, representing a prevalence of 0.14% [95% confidence interval (CI) 0.10, 0.19]. Patients were predominantly male (77.1%) with a median age of 58 years (interquartile range [IQR] 52.5, 68.0). Acute myocardial infarction (42.9%) was the most common aetiology of CS, followed by cardiac arrhythmias (20.0%). Endoscopic retrograde cholangiopancreatography (ERCP) was performed in 77.1% of cases after a median of 33 days following CS onset [IQR 24, 65], showing typical biliary casts (60.0%), intraductal filling defects (28.6%), and bile duct obliteration (20.0%). Cast removal and stent placement was performed in nearly half of ERCP procedures (45.7%). Magnetic resonance cholangiopancreatography (MRCP) was performed in 22.9% of cases and showed intraductal dilation (11.4%), lumen narrowing (17.1%), or strictures (14.3%). Median intensive care unit and hospital length of stay was 43 days [IQR 33, 66] and 58 days [IQR 33, 88], respectively. In‐hospital mortality was 57.1%. One‐year (65.7%) and 3‐year (71.4%) mortality remained high. Two patients underwent liver transplantation after a median of 113 days [IQR 105, 122] and were alive at 3‐year follow‐up.

**Conclusions:**

In this multicentre retrospective analysis in a high‐risk CS cohort, SSC‐CIP was a rare yet serious complication of intensive care unit stay with high in‐hospital mortality. Treatment options are limited, and liver transplantation remains the only viable long‐term treatment option.

## Introduction

Liver dysfunction in cardiogenic shock (CS) is common and is associated with increased mortality.^1^ In critically ill patients, hypoxic hepatitis must be differentiated from cholestatic liver injury due to ischemic cholangiopathy, termed ‘secondary sclerosing cholangitis in critically ill patients’ (SSC‐CIP), a rare progressive syndrome of rapid bile duct destruction and subsequent liver failure. SSC‐CIP has primarily been recognized in critically ill populations experiencing trauma, burns, or acute respiratory distress syndrome, yet the underlying pathogenesis remains unclear.^2^, ^3^, ^4^, ^5^ As opposed to hepatocytes, which receive dual blood supply from the portal vein and hepatic artery, biliary epithelium uniquely relies solely on perfusion from the peribiliary plexus of hepatic artery branches.^5^, ^6^ In SSC‐CIP, low perfusion states and biliary ischaemia are hypothesized to contribute to bile necrosis and development of typical biliary casts, identified mainly through endoscopic retrograde cholangiopancreatography (ERCP) and less commonly magnetic resonance cholangiopancreatography (MRCP).^7^ Evidence supporting this hypothesis of ischaemia to vulnerable intrahepatic biliary epithelium is lacking. Potential risk factors for the development of SSC‐CIP such as high‐dose vasopressors, positive pressure mechanical ventilation, ketamine use, toxic bile composition, or biliary infections have been discussed.^5^ Prognosis of SSC‐CIP is poor, and liver transplantation often represents the only long‐term treatment option.^2^, ^3^ Further, CS patients suffer from severe organ hypoperfusion,^8^ yet the incidence of SSC‐CIP in this critically ill population is poorly described. Given the limited evidence and severity of this syndrome, we aimed to further investigate SSC‐CIP in the context of CS.

## Methods

24 251 total CS patients admitted between 1 January 2010 and 31 December 2023 were retrospectively screened for the diagnosis of SSC‐CIP across nine German tertiary care centers. Patients in which SSC‐CIP was suspected though not confirmed were excluded from analysis following a critical internal review of individual cases. Following identification of CS patients with a diagnosis of SSC‐CIP, chart review of baseline characteristics, laboratory values, SSC‐specific imaging, diagnostics, and outcomes as per pre‐selected variables was performed.

### Diagnostic criteria for secondary sclerosing cholangitis in critically ill patients

In patients with suspected SSC‐CIP, MRCP and ERCP are considered gold‐standard diagnostic imaging modalities. While guideline recommendations specifying mandatory imaging criteria for the diagnosis do not exist, diagnosis of SSC‐CIP was confirmed in our cohort by the presence of the following: (I) ribbon‐like intraductal filling defects, (II) biliary casts, (III), biliary strictures and/or bile duct dilation, or (IV) obliteration of bile ducts (pruned‐tree appearance).^3^, ^4^, ^5^ While ultrasound may provide evidence of intrahepatic or extrahepatic biliary dilation and complications of SSC‐CIP, findings are not sufficiently specific and were not used to confirm SSC‐CIP.

### Statistical analysis

All statistical analyses were performed using R**®** (version 4.2.2, The R foundation, Vienna, Austria). Continuous variables are reported as medians and interquartile ranges (25th and 75th). Categorical variables are reported as absolute values and percentages. Characteristics of included patients were compared using Wilcoxon rank‐sum tests for continuous variables. Categorical variables were compared using Fisher's exact or chi‐square test. All tests were two‐tailed, and *P*‐values <0.05 were considered significant.

## Results

### Patient characteristics and intensive care unit management

Thirty‐five CS patients with a diagnosis of SSC‐CIP were included, representing a prevalence of 0.14% [95% confidence interval (CI) 0.10, 0.19]. Patients were predominantly male (77.1%) with a median age of 58 years (interquartile range [IQR] 52.5, 68.0). Acute myocardial infarction (42.9%) was the most common aetiology of CS, followed by cardiac arrhythmias (20.0%). Median SAPS II score at admission was 62.0 [IQR 54.8, 69.8], and 54.3% of patients had experienced cardiac arrest. Vasopressor requirement was high, and nearly half of the patients underwent venoarterial extracorporeal membrane oxygenation (VA‐ECMO) support (48.6%) (*Table* *1*). Ursodeoxycholic acid (UDCA) was used in 27/35 patients and there was no difference in length of stay or mortality outcome between those with and without treatment (*Table* *S1*). The *P*‐values should, however, be interpreted with caution given that the present study was not specifically designed to assess the efficacy of ursodeoxycholic acid therapy.

**Table 1:** Baseline characteristics and intensive care unit management.

Characteristics		SSC‐CIP (*n* = 35)
Demographics		
Age [years], median [IQR]		58.0 [52.5, 68.0]
Sex [male], *n* (%)		27 (77.1)
Body mass index [kg/m^2^], median [IQR]		27.9 [24.0, 31.2]
ICU characteristics		
SAPS II score, median [IQR]		62.0 [54.8, 69.8]
Cardiac arrest, *n* (%)		19 (54.3)
Extracorporeal cardiopulmonary resuscitation, *n* (%)		9 (25.7)
VA‐ECMO, *n* (%)		17 (48.6)
Impella therapy, *n* (%)		6 (17.1)
Mechanical ventilation, *n* (%)		34 (97.1)
Renal replacement therapy, *n* (%)		23 (65.7)
Vasopressors	Epinephrine, *n* (%)	12 (34.3)
	Norepinephrine, *n* (%)	35 (100.0)
	Dobutamine, *n* (%)	18 (51.4)
	Vasopressin, *n* (%)	16 (45.7)
Type of cardiogenic shock	ST‐elevation myocardial infarction, *n* (%)	12 (34.3)
	Non‐ST segment elevation myocardial infarction, *n* (%)	3 (8.6)
	Cardiomyopathy, *n* (%)	5 (14.3)
	Myocarditis, *n* (%)	5 (14.3)
	Cardiac arrhythmia, *n* (%)	7 (20.0)
	Pulmonary embolism, *n* (%)	0 (0.0)
	Others, *n* (%)	3 (8.6)

ICU, intensive care unit; IQR, interquartile range; *n*, number; SAPS, simplified acute physiology score; SSC‐CIP, secondary sclerosing cholangitis in critically ill patients; VA‐ECMO, venoarterial extracorporeal membrane oxygenation.

### Secondary sclerosing cholangitis in critically ill patients' diagnostics

ERCP was performed in 77.1% of cases after a median of 33 days following CS onset [IQR 24, 65], showing typical biliary casts (60.0%), intraductal filling defects (28.6%), and bile duct obliteration (20.0%). Cast removal and bile stent placement were performed in nearly half of ERCP procedures (45.7%). Only a minority (20.0%) of patients showed bacterial growth on bile cultures. MRCP was performed in 22.9% of cases and showed intraductal dilation (11.4%), lumen narrowing (17.1%), or strictures (14.3%). Abdominal ultrasound was performed in 91.4% of cases after a median of 20 days in the intensive care unit (ICU) [IQR 15.7, 61.0], and intrahepatic (14.3%) and extrahepatic (11.4%) bile duct dilation was not a common finding. Patients showed typical elevation of liver values during ICU stay (*Table* *2*).

**Table 2:** Secondary sclerosing cholangitis in critically ill patients' diagnostics.

Characteristics	SSC‐CIP (n = 35)
Liver‐specific laboratory values in ICU	
Peak bilirubin [mg/dL], median [IQR]	17.3 [10.9, 26.1]
Peak alkaline phosphatase [U/L], median [IQR]	991 [349, 1417]
Peak aspartate aminotransferase [U/I], median [IQR]	960 [457, 2580]
Peak alanine aminotransferase [U/I], median [IQR]	680 [344, 1560]
Peak gamma‐glutamyl transferase [U/I], median [IQR]	1037 [485, 1608]
ERCP	
ERCP for SSC‐CIP performed, *n* (%)	27 (77.1)
Time from ICU admission to first ERCP [d], median [IQR]	33 [24, 65]
Evidence of ribbon‐like intraductal defects, *n* (%)	10 (28.6)
Evidence of biliary casts, *n* (%)	21 (60.0)
Evidence of biliary strictures and/or dilation beyond second bifurcation of intrahepatic bile ducts, *n* (%)	12 (34.3)
Evidence of total obliteration of bile ducts (pruned‐tree appearance), *n* (%)	7 (20.0)
Removal of biliary casts, *n* (%)	16 (45.7)
Balloon dilation, *n* (%)	4 (11.4)
Stent implantation, *n* (%)	16 (45.7)
Placement of nasobiliary drainage, *n* (%)	3 (8.6)
Positive bile culture, *n* (%)	7 (20.0)
MRCP	
MRCP for SSC‐CIP performed, *n* (%)	8 (22.9)
Time from ICU admission to first MRCP [d], median [IQR]	75 [47, 125]
Intrahepatic bile duct dilation, *n* (%)	4 (11.4)
Evidence of hypoechoic wall thickening or lumen narrowing of intrahepatic bile ducts, *n* (%)	6 (17.1)
Evidence of extrahepatic biliary strictures, *n* (%)	2 (5.7)
Evidence of biliary strictures and/or dilation beyond second bifurcation of intrahepatic bile ducts, *n* (%)	5 (14.3)
Abdominal ultrasound	
Ultrasound for SSC‐CIP performed, *n* (%)	32 (91.4)
Time from ICU admission to first ultrasound for SSC‐CIP [d], median [IQR]	20.0 [15.7, 61.0]
Liver parenchyma with atrophy, *n* (%)	1 (2.9)
Liver parenchyma with hypertrophy, *n* (%)	9 (25.7)
Liver parenchyma with cirrhotic morphology, *n* (%)	3 (8.6)
Intrahepatic bile duct dilation, *n* (%)	5 (14.3)
Extrahepatic bile duct dilation, *n* (%)	4 (11.4)
Hepatic artery thrombosis, *n* (%)	0 (0.0)
Biloma, *n* (%)	0 (0.0)
Evidence of hypoechoic wall thickening or lumen narrowing of intrahepatic bile ducts, *n* (%)	3 (8.6)
Signs of cholecystitis, *n* (%)	1 (2.9)
Signs of hepatolithiasis, n (%)	1 (2.9)
Signs of choledocholithiasis, *n* (%)	4 (11.4)
Bile sludge, *n* (%)	6 (17.1)
Evidence of cholangiocellular carcinoma, *n* (%)	0 (0.0)
Gallbladder perforation, *n* (%)	0 (0.0)
Signs of portal hypertension including splenomegaly, portosystemic collaterals, and ascites, *n* (%)	8 (22.9)
Portal lymphadenopathy, *n* (%)	1 (2.9)

D, days; ERCP, endoscopic retrograde cholangiopancreatography; ICU, intensive care unit; IQR, interquartile range; MRCP, magnetic resonance cholangiopancreatography; SSC‐CIP, secondary sclerosing cholangitis in critically ill patients.

### Outcomes

Median ICU and hospital length of stay was 43 days [IQR 33, 66] and 58 days [IQR 33, 88], respectively. In‐hospital mortality was 57.1%. One‐year (65.7%) and 3‐year (71.4%) mortality remained high. Two patients underwent liver transplantation after a median of 113 days [IQR 105, 122] and were alive at the 3‐year follow‐up (*Table* *3*).

**Table 3:** Secondary sclerosing cholangitis in critically ill patients' treatment and outcomes.

Characteristics	SSC‐CIP (*n* = 35)
SSC‐CIP specific medication	
Ursodeoxycholic acid treatment, *n* (%)	27 (77.1)
Outcome	
Total ICU length of stay [d], median [IQR]	43 [33, 66]
Total hospital length of stay [d], median [IQR]	58 [33, 88]
Hospital mortality, *n* (%)	20 (57.1)
1‐year mortality, *n* (%)	23 (65.7)
3‐year mortality, *n* (%)	25 (71.4)^a^
Liver transplantation due to SSC‐CIP	
LTx due to SSC‐CIP performed, *n* (%)	2 (5.7)
MELD score at time of listing for LTx, median [IQR]	29.5 [28.8,30.3]
MELD score at time of LTx, median [IQR]	33.5 [32.8,34.3]
Time from ICU admission to listing for LTx [d], median [IQR]	82 [78, 86]
Time from ICU admission to LTx [d], median [IQR]	113 [105, 122]
Early complications after LTx (<1 year), *n* (%)	0 (0.0)
Late complications after LTx (>1 year), *n* (%)	0 (0.0)
Hospital mortality after LTx, *n* (%)	0 (0.0)
1‐year mortality after LTx, *n* (%)	0 (0.0)
3‐year mortality after LTx, *n* (%)	0 (0.0)

D, days; ICU, intensive care unit; *n*, number; IQR, interquartile range; LTx, liver transplantation; MELD, model of end‐stage liver disease; SSC‐CIP, secondary sclerosing cholangitis in critically ill patients.

^a^ A total of nine patients were lost to 3‐year follow‐up.

## Discussion

This is the first study to report on the incidence of SSC‐CIP in the context of CS. SSC‐CIP was a rare but serious complication with a prevalence of 0.14% in a cohort of 24 251 CS patients. True prevalence of SSC‐CIP in the critically ill is unclear, as case numbers often reported from liver transplant centers are subject to selection bias.^9^, ^10^ Further, the observed in‐hospital mortality was higher than reported in most CS cohorts, though rates were consistent with findings from previously reported SSC‐CIP studies.^3^, ^4^ Despite extensive length of stay in the ICU, most SSC‐CIP diagnoses were made after 1 month using ERCP, reflecting the difficulties of establishing the diagnosis. While liver values rise quickly in CS and development of hypoxic hepatitis is common,^11^ early clinical suspicion of SSC‐CIP in patients with increased cholestatic laboratory values should prompt timely consultation with gastroenterology. Leonhardt *et al*. found a cholestatic injury on laboratory analysis after a median of 7 days in a cohort of 16 SSC‐CIP patients with a median ICU stay of 31 days.^9^ Confirmation of SSC‐CIP diagnosis has been reported in cohorts following a median of 44^4^ and 89 days^2^ in the ICU. This delay in diagnosis may be explained by reluctance to perform invasive diagnostic imaging in haemodynamically unstable patients, and frequent re‐evaluation of optimal ERCP timing should follow interdisciplinary discussion. Further, high ERCP utilization indicates this may be the diagnostic tool of choice, though interventional treatment with bile cast removal has not shown to improve prognosis. Removal of bile casts and stent implantation with ERCP in nearly half of SSC‐CIP cases proved a safe procedure, with only 5.7% developing cholangiosepsis in our cohort. Though reports of improved biliary drainage and drop in cholestatic laboratory values following ERCP intervention suggest short‐term relief,^6^, ^12^ progressive biliary destruction leading to cirrhosis does not seem to be halted. Liver transplantation is the only viable treatment option should SSC‐CIP progress to biliary cirrhosis, and early evaluation of transplant candidacy is essential. Only two patients (5.7%) in our cohort underwent successful liver transplantation for SSC‐CIP and were alive after 3 years, making comparison with other cohorts difficult. Observed low liver transplantation rates and high mortality in CS reflect poor transplant candidacy and lack of feasible treatment options in these patients. CS patients may be particularly vulnerable to a rapid and severe course of SSC‐CIP, especially in the context of low‐perfusion states and the need for mechanical circulatory support. SSC‐CIP patients lack prior history of liver disease or biliary obstruction, and rapid development of biliary cirrhosis and liver failure in relatively young and previously healthy individuals calls for further research into risk factors and treatment options to improve exorbitantly high mortality rates.

### Limitations

Though many CS patients were screened, not all SSC‐CIP patients may have been identified, for example, due to a lack of diagnostics or death prior to diagnosis, representing a potential limitation of our study. Further, this cohort of severely ill patients suffered common complications of a long ICU stay, yet our work cannot comment on previously discussed risk factors for development of SSC‐CIP due to lack of a comparison group.

## Conclusions

Long duration of ICU stay and high in‐hospital mortality in SSC‐CIP should encourage early suspicion of disease in patients suffering CS and cholestatic liver injury, triggering a timely diagnostic workup to identify potential transplant candidates prior to development of biliary cirrhosis. Future studies should aim to improve understanding of triggering factors und management of SSC‐CIP to improve outcomes.

## Conflict of interest

The authors declare no conflict of interest.

## Funding

There was no funding for this study.

## Supporting information


**Table S1.** Ursodeoxycholic acid treatment.

## Data Availability

The data are not publicly available due to ethical restrictions and legal constraints. Readers may contact the corresponding author for reasonable requests for the data. De‐identified data may be provided after approval from the ethical review board.

## References

[1] Jung C , Fuernau G , Eitel I , Desch S , Schuler G , Kelm M , *et al*. Incidence, laboratory detection and prognostic relevance of hypoxic hepatitis in cardiogenic shock. Clin Res Cardiol 2017;106:341‐349. doi:10.1007/s00392-016-1060-3 27928583

[2] Weig T , Schubert MI , Gruener N , Dolch ME , Frey L , Miller J , *et al*. Abdominal obesity and prolonged prone positioning increase risk of developing sclerosing cholangitis in critically ill patients with influenza A‐associated ARDS. Eur J Med Res 2012;17:30. doi:10.1186/2047-783X-17-30 23259907 PMC3543205

[3] Ben‐Ari Z , Levingston D , Weitzman E , Haviv‐Yadid Y , Cohen‐Ezra O , Weiss P , *et al*. Secondary sclerosing cholangitis following major burn. Ann Hepatol 2015;14:695‐701. doi:10.1016/S1665-2681(19)30764-1 26256898

[4] Voigtländer T , Negm AA , Schneider AS , Strassburg CP , Manns MP , Wedemeyer J , *et al*. Secondary sclerosing cholangitis in critically ill patients: model of end‐stage liver disease score and renal function predict outcome. Endoscopy 2012;44:1055‐1058. doi:10.1055/s-0032-1325733 23108773

[5] Leonhardt S , Veltzke‐Schlieker W , Adler A , Schott E , Hetzer R , Schaffartzik W , *et al*. Trigger mechanisms of secondary sclerosing cholangitis in critically ill patients. Crit Care 2015;19:131. doi:10.1186/s13054-015-0861-5 25886728 PMC4407292

[6] Gelbmann CM , Rümmele P , Wimmer M , Hofstädter F , Göhlmann B , Endlicher E , *et al*. Ischemic‐like cholangiopathy with secondary sclerosing cholangitis in critically ill patients. Am J Gastroenterol 2007;102:1221‐1229. doi:10.1111/j.1572-0241.2007.01118.x 17531010

[7] Gudnason HO , Björnsson ES . Secondary sclerosing cholangitis in critically ill patients: current perspectives. Clin Exp Gastroenterol 2017;10:105‐111. doi:10.2147/CEG.S115518 28694703 PMC5491618

[8] van Diepen S , Katz JN , Albert NM , Henry TD , Jacobs AK , Kapur NK , *et al*. Contemporary management of cardiogenic shock: a scientific statement from the American Heart Association. Circulation 2017;136:e232‐e268. doi:10.1161/CIR.0000000000000525 28923988

[9] Leonhardt S , Veltzke‐Schlieker W , Adler A , Schott E , Eurich D , Faber W , *et al*. Secondary sclerosing cholangitis in critically ill patients: clinical presentation, cholangiographic features, natural history, and outcome: a series of 16 cases. Medicine (Baltimore) 2015;94:e2188. doi:10.1097/MD.0000000000002188 26656347 PMC5008492

[10] Voigtländer T , Jaeckel E , Lehner F , Manns MP , Lankisch TO . Liver transplantation for critically ill patients with secondary sclerosing cholangitis: outcome and complications. Liver Transpl 2015;21:1295‐1299. doi:10.1002/lt.24192 26069199

[11] Jäntti T , Tarvasmäki T , Harjola VP , Parissis J , Pulkki K , Sionis A , *et al*. Frequency and prognostic significance of abnormal liver function tests in patients with cardiogenic shock. Am J Cardiol 2017;120:1090‐1097. doi:10.1016/j.amjcard.2017.06.049 28821350

[12] Jaeger C , Mayer G , Henrich R , Gossner L , Rabenstein T , May A , *et al*. Secondary sclerosing cholangitis after long‐term treatment in an intensive care unit: clinical presentation, endoscopic findings, treatment, and follow‐up. Endoscopy 2006;38:730‐734. doi:10.1055/s-2006-925241 16874910

