# Supplementary material for: Secondary sclerosing cholangitis in patients suffering cardiogenic shock
Source: ESC Heart Fail. 2025 Feb 26;12(3):2239–44. doi: 10.1002/ehf2.15248 (PMC12055343; doi:10.1002/ehf2.15248)
Supplement: Supplementary file 1 — Table S1. Ursodeoxycholic acid treatment. [file EHF2-12-2239-s001.docx]

**- Supplementary Tables -**

| **Supplementary Table 1. Ursodeoxycholic acid treatment** | | | |
| --- | --- | --- | --- |
| **Characteristics** | **UDCA treatment**  **(n = 27)** | **No UDCA treatment**  **(n = 8)** | **p-value** |
| **Outcome** | | | |
| Total ICU length of stay [d], median [IQR] | 43.00 [32.50, 66.00] | 40.50 [32.25, 61.75] | 0.922 |
| Total hospital length of stay [d], median [IQR] | 64.00 [33.00, 87.50] | 40.50 [32.25, 61.75] | 0.455 |
| Hospital mortality, n (%) | 16 (59.3) | 4 (50.0) | 0.700 |
| 1-year mortality, n (%) | 18 (66.7) | 5 (62.5) | >0.999 |
| 3-year mortality*, n (%) | 20 (74.1) | 5 (62.5) | >0.999 |
| LTx due to SSC-CIP performed, n (%) | 2 (7.4) | 0 (0.0) | >0.999 |

**Supplementary Table 1. Ursodeoxycholic acid treatment.** UDCA, Ursodeoxycholic acid; ICU, intensive care unit; IQR, interquartile range; SSC-CIP, secondary sclerosing cholangitis in critically ill patients; LTx, liver transplantation. *A total of nine patients were lost to 3-year follow-up.
